# Nanofibrous PEDOT-Carbon Composite on Flexible Probes for Soft Neural Interfacing

**DOI:** 10.3389/fbioe.2021.780197

**Published:** 2021-11-26

**Authors:** Venkata Suresh Vajrala, Valentin Saunier, Lionel G. Nowak, Emmanuel Flahaut, Christian Bergaud, Ali Maziz

**Affiliations:** ^1^ Laboratory for Analysis and Architecture of Systems (LAAS), CNRS, Toulouse, France; ^2^ Centre de Recherche Cerveau et Cognition (CerCo), CNRS, Toulouse, France; ^3^ CIRIMAT, CNRS, Université de Toulouse, Toulouse, France

**Keywords:** PEDOT-Carbon, carbon nanofibers, porous composite, flexible neural interfaces, electrophysiological recording, neural stimulation

## Abstract

In this study, we report a flexible implantable 4-channel microelectrode probe coated with highly porous and robust nanocomposite of poly (3,4-ethylenedioxythiophene) (PEDOT) and carbon nanofiber (CNF) as a solid doping template for high-performance *in vivo* neuronal recording and stimulation. A simple yet well-controlled deposition strategy was developed *via in situ* electrochemical polymerization technique to create a porous network of PEDOT and CNFs on a flexible 4-channel gold microelectrode probe. Different morphological and electrochemical characterizations showed that they exhibit remarkable and superior electrochemical properties, yielding microelectrodes combining high surface area, low impedance (16.8 ± 2 MΩ µm^2^ at 1 kHz) and elevated charge injection capabilities (7.6 ± 1.3 mC/cm^2^) that exceed those of pure and composite PEDOT layers. In addition, the PEDOT-CNF composite electrode exhibited extended biphasic charge cycle endurance and excellent performance under accelerated lifetime testing, resulting in a negligible physical delamination and/or degradation for long periods of electrical stimulation. *In vitro* testing on mouse brain slices showed that they can record spontaneous oscillatory field potentials as well as single-unit action potentials and allow to safely deliver electrical stimulation for evoking field potentials. The combined superior electrical properties, durability and 3D microstructure topology of the PEDOT-CNF composite electrodes demonstrate outstanding potential for developing future neural surface interfacing applications.

## 1 Introduction

Neural electrodes provide the critical interface between the nervous system and electronics. Well-defined anatomical regions from the brain can be the targets of implanted microelectrodes, enabling localized neuromodulation by either recording or delivering electrical signals at the level of individual neuron (Nicolelis et al., 2003; Spira and Hai 2013). Such capabilities have been critically important for supporting neuroscience research along with emerging clinical devices aimed at treating debilitating disorders, including deafness (Sparreboom et al., 2010), paralysis (Schultz and Kuiken 2011), blindness (Rizzo et al., 2003), Parkinson’s disease (Benabid 2003), epilepsy (Theodore and Fisher 2004) and other disorders (Mayberg et al., 2005). In all of these applications, the crucial material-dependent problem is developing microelectrode array that sense and/or stimulate neural activity from small, targeted groups of neurons with high fidelity and long-term reliability (Grill et al., 2009).

Conventional implantable microelectrode arrays, made of silicon backbone and noble metal electrodes, such as gold (Au) platinum (Pt) or iridium (Ir) become routine in animal research and have occasionally been used in humans (Khodagholy et al., 2015; Kellis et al., 2016; Jun et al., 2017). However, they are not suitable for long-term use due to limitations regarding the electrical and mechanical mismatches with the surrounding tissue (Polikov et al., 2005). Decreasing the size of an electrode active site, to ideally target single neuron, results in low capacitance and high impedance at the electrode/tissue interface, which seriously impacts recording resolution and stimulation capabilities (Cogan 2008; Viswam et al., 2019). For neuronal recording, the electrode impedance contributes to the noise, and high impedance electrodes are expected to have a low signal-to-noise ratio (SNR) (Boehler et al., 2020). For neuronal stimulation, an ideal electrode should display a high storage capability to safely inject current pulses with minimal potential transients at the electrode/tissue interface, thus decreasing both electrode polarization and heat accumulation during stimulation (Merrill et al., 2005; Cogan 2008; Boehler et al., 2020).

Besides, immune reaction occurs through the mechanical mismatch between rigid electrodes and the neural tissue, triggering inflammatory responses and glial scar formation, which may lead to encapsulation of the electrodes and subsequent device failure (Polikov et al., 2005; Jeong et al., 2015). These device failures appear in the form of electrical recording degradations including increased impedance, increased noise levels and decreased signal amplitudes (Jeong et al., 2015). In this regard, there has been a demand to fabricate electrodes on flexible substrates that, by showing smaller hardness mismatch, provide a more adaptable interface to neural tissue. In addition, the electrode material deposited on flexible substrate should display low electrical impedance and high charge-transfer capacity without substantially increasing the site geometric surface area (Inal et al., 2018).

To tackle this challenge, various types of organic electroactive materials have been employed such as conductive polymers (CPs) (Green and Abidian 2015; Maziz et al., 2021), carbon-based nanomaterials *i.e.*, carbon nanotubes (CNTs) (Ansaldo et al., 2011; Hanein and Bareket-Keren 2013; Sridharan and Muthuswamy 2021), graphene (Hess et al., 2011), reduced graphene oxide (r-GO) (Luo et al., 2013) and their nanocomposites (Gerwig et al., 2012; Saunier et al., 2020b), to create much desired porosity and softness at the electrode/tissue interface. Among them, the conducting polymer Poly (3,4-ethylenedioxythiophene) (PEDOT) has been a popular choice due to its mixed electronic and ionic conductivities, high-quality electrochemical performances, together with excellent biocompatibility, softness, and ease of functionalization (Abidian et al., 2009; Fabretto et al., 2012; Green and Abidian 2015; Maziz et al., 2016). Several reports showed that PEDOT coatings, doped with different counter ions such as poly (styrene sulfate) (PSS), Nafion, tosylate (Larsen et al., 2012; Maziz et al., 2017), dodecyl sulfate (Wu et al., 2015) or ClO_4_^−^(Maziz et al., 2014), can significantly decrease the electrode impedance (30–250 MΩ/µm^2^) and increase the charge-injection capacity (1–3 mC/cm^2^) as compared to flat metal sites of similar geometric area (Cogan 2008; Venkatraman et al., 2009; Khodagholy et al., 2011). In addition, *In vitro* studies have demonstrated that PEDOT coating would also present a good substrate for the growth of various cell types in biological and tissue engineering areas, wherein PEDOT directly interacts with cells or tissues (Bongo et al., 2013). Despite its promising outlook, PEDOT is yet to be perfected as a coating material for neural electrodes, especially in terms of extended charge injection capabilities and long-term adhesion stability (Cui and Zhou 2007; Jan et al., 2009). For example, brain stimulation applications demand high and stable charge injection capabilities, where the electrode materials should sustain few thousands to millions of cycles of electrical stimulations pulses without corrosion, tissue damage, or delamination (Boehler et al., 2020).

Multiple strategies have been suggested to reinforce PEDOT coating, either by modifying the monomer itself (Ouyang et al., 2017), using adhesion promoters (Boehler et al., 2017), or by the incorporation of charged carbonaceous nanomaterials (Luo et al., 2011; Gerwig et al., 2012). In previous studies, composite materials made up of CNTs (Bhandari et al., 2009; Gerwig et al., 2012) or r-GO (Luo et al., 2013), in combination with PEDOT, have been coated on metal microelectrodes to decrease the impedance and ramp-up the charge injection limits (Samba et al., 2015), even beyond the PEDOT: PSS capabilities for both acute and chronic stimulation tests (Alba et al., 2015; Kozai et al., 2016). It was also reported that these mechanically strong carbonaceous materials function as reinforcing elements within the composite, preventing the PEDOT film from undergoing deformation and cracking during prolonged redox reactions (Luo et al., 2011). This excellent performance makes the combination of PEDOT with carbon-based nanomaterials a highly promising candidate material for the development of long-lasting neural interfaces. However, as of now, most of the research has focused on either the selection of near perfect electrode material with superior electrical properties or controlled 3D surface macro porous pattering of electrode to promote an intimate contact with the neural tissue. In contrast, less attention has been paid to the combination of both.

Recently, we have demonstrated the feasibility of a novel composite material by combining PEDOT with carbon nanofibers (CNFs) through a simple and reproducible electrodeposition method (Saunier et al., 2020b). CNFs exhibit extraordinary strength, high modulus of elasticity (940 GPa), and provide an extremely large surface area for charge transfer and cell attachment (Nguyen-Vu et al., 2006). Since, they contain basal graphite planes and edge planes, upon oxidation, their outer surface can be electrochemically functionalized with PEDOT molecules. In addition, as a result of their extremely high edge proportion with very high aspect ratios and inherent herringbone morphology, making them excellent nanoscale building block to establish interconnected, three-dimensional (3D) macroporous structures, in combination with PEDOT. We have recently shown that the combination of CNFs and PEDOT on rigid microelectrode array (MEA) resulted in as strong synergetic effect between the two components in the single composite leading to remarkable electrochemical properties, as well as a reliable *in vitro* neurotransmitter monitoring using amperometric techniques (Saunier et al., 2020b). These results suggest PEDOT-CNF composites as a most interesting electrode material for applications in neuroprostheses and neurophysiology research. In this context, to go further into the development of neural interfacing devices for *in vitro* and *in vivo* applications, we are reporting a method for preparing macroporous, stable and electrically superior PEDOT, using CNFs as a solid dopant template, on ultra-flexible penetrating neural microelectrodes. We developed a well-controlled single step deposition method and optimized it for preparing macroporous PEDOT-CNFs nanocomposite *via in situ* electrochemical polymerization technique on flexible parylene-based neural probes. This flexible substrate-provides the means to decrease the mechanical mismatch at the electrode/tissue interface. We showed that PEDOT-CNF hybrid neural microelectrodes exhibit remarkable electrochemical properties, yielding microelectrodes combining low impedance, high surface area, and elevated charge injection capabilities. This device was further tested for neural recording and stimulation in the hippocampus of mouse brain slice *in vitro*. The obtained results opened great prospects for the development of next-generation microelectrodes for applications in brain therapies.

## 2 Materials and Methods

### 2.1. Fabrication of the Neural Implant and Device Packaging

**FIGURE 1:**
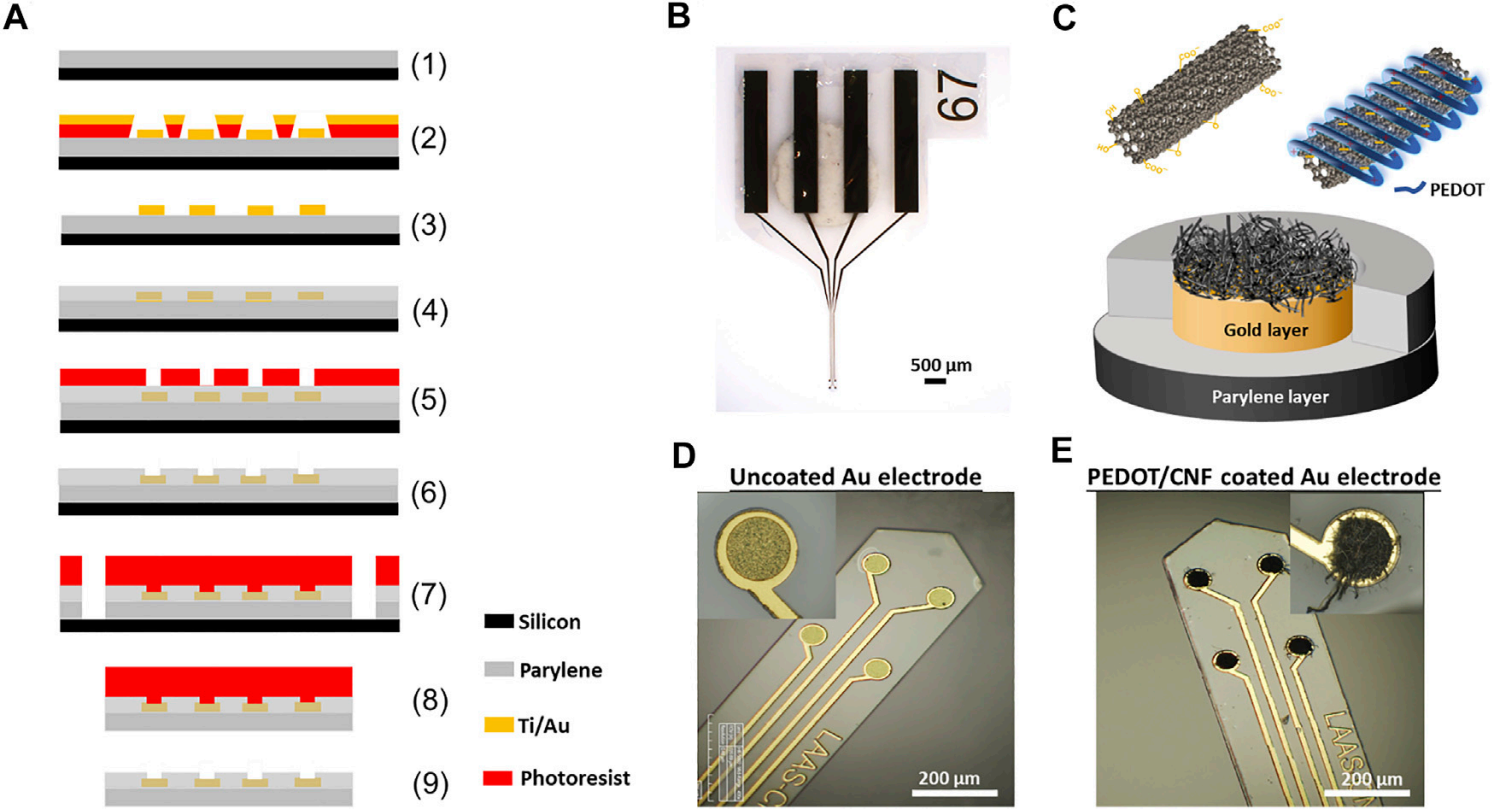
Schematic illustration and optical images of neural implant. **(A)** Schematic overview of steps involved in the microfabrication procedure- (1) 23 µm thick Parylene C deposition on SiP wafer; (2) Photo-patterning of nLof and gold layer deposition (Ti/Au-50/200 nm); (3) nLof removal; (4) 2nd Parylene C layer (1.3 µm) deposition and anneal at 110°C for 16 h; (5) Spin coating of 5 µm thick AZ4562 photoresist; (6) RIE with ICP-RIE oxygen plasma; (7) Photo-patterning with BPN photoresist and RIE with ICP-DRIE oxygen plasma; (8) Stripping of implants from SiP wafer and (9) Development with NF52 and cleaning with DI water. **(B)** Optical micrograph of the neural implant. **(C)** Schematic representation of the PEDOT-CNF composite deposition. **(D)** and **(E)** Optical micrographs showing the neural microelectrodes before and after surface modification with PEDOT-CNF composite.

The photomask designs of the flexible parylene C-based probe, with four micro disk electrodes, are inspired from our previous works (Castagnola et al., 2015; Lecomte et al., 2017). The fabrication procedure is schematically illustrated in Figure 1A. A 23 µm thick film of Parylene C was deposited using chemical vapour deposition (Comelec C-30-S at 700°C) on 4-inch SiP wafer. Next 50 nm thick Ti and 200 nm thick Au layers were deposited and patterned, using a conventional physical vapour deposition technique followed by a lift-off with AZ-nLof 2035 (Micro chemicals). Subsequently, 1.3 µm thick parylene C was deposited as a passivation layer, followed by an annealing step at 110°C for 16 h under nitrogen flow to increase the adhesion between parylene C-gold sandwich layers. Next, the electrode surfaces (40 µm diameter) and the corresponding connection pads were realized by, photo-patterning with 5 µm thick AZ4562 photoresist, followed by the etching of the thin parylene C passivation layer using O_2_ plasma reactive ion etching ICP-RIE (Trikon Omega 201). Later, the implants were anistropically etched to establish smooth outlines and vertical sidewalls, using 50 µm thick BPN photoresist (Intervia BPN-65A, Dupont) and a deep reactive ion etching (ICP-DRIE) step. After that, the implants were peeled-off carefully by placing the entire wafer into DI water for at least 2 h. Later, the released implants were stripped off from the leftover photoresist using TechniStrip NF52 (Microchemicals), thoroughly washed in DI water and stored in a dry place. Finally, the implants were bonded to a customized flexible ribbon cable with golden traces (AXO-00021, pro-POWER, China) by using epoxy silver and photosensitive glue.

### 2.2. Functionalization of Carbon Nanofibers

Raw CNFs (Pyrograf®-III, PR-19-XT-PS, pyrolytically stripped, platelets conical, >98% carbon basis, 20–200 µm) were oxidized using wet chemical oxidation process where 300 mg of CNFs were placed in 200 ml of the oxidizing solution (15 M) HNO_3_/(18 M) H_2_SO_4_ and sonicated for 15 min, followed by 2 h of reflux at 70°C (Rasheed et al., 2007; Bortolamiol et al., 2014). This process helps to remove impurities (metallic particles and amorphous carbon) from the sample and make them hydrophilic, so that the CNFs can be dispersed in water (Saunier et al., 2020a). Following the acid treatment, the CNF dispersion was washed with DI water to reach a neutral pH, and stored at 4°C.

### 2.3. Electrochemical Deposition of PEDOT-CNF Composite

**FIGURE 2:**
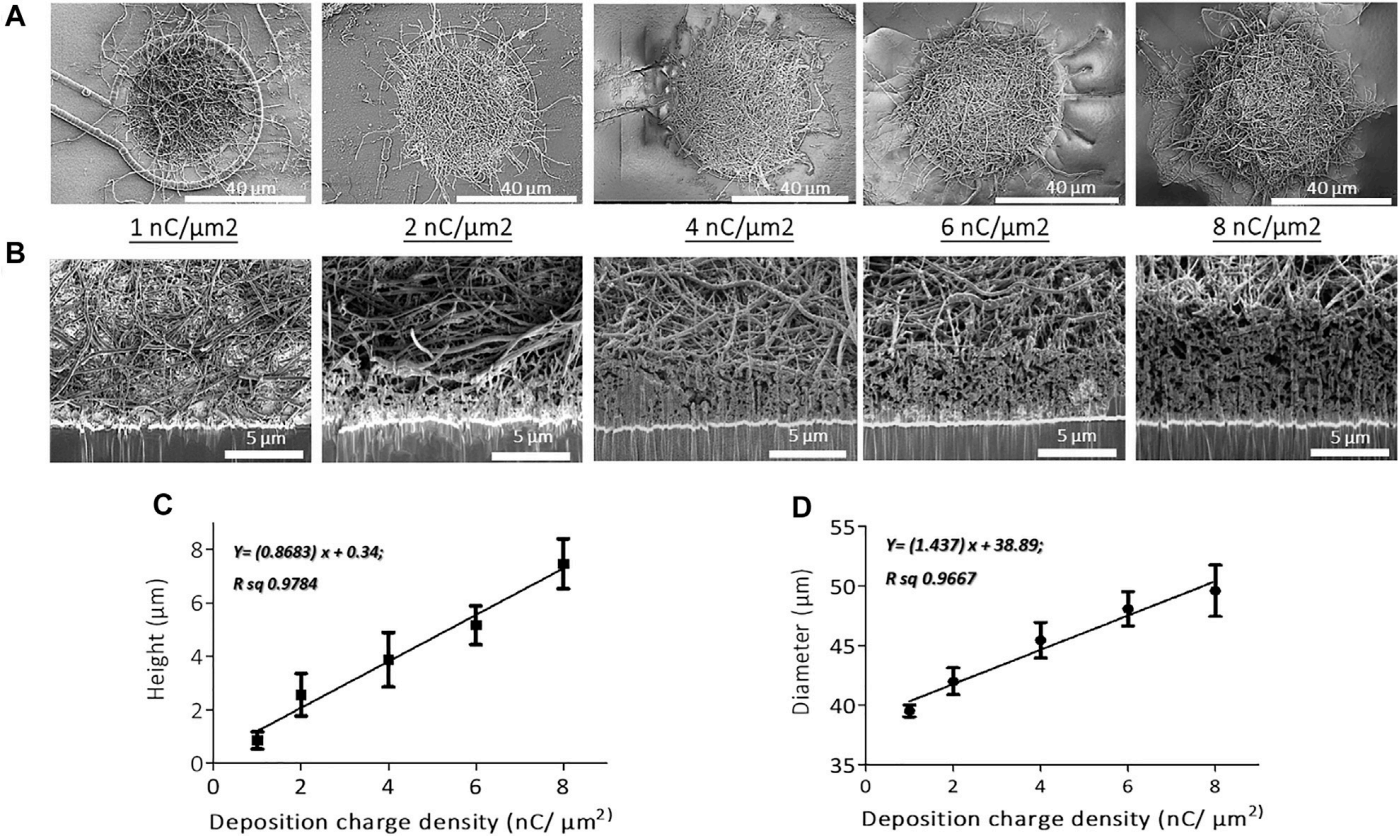
SEM micrographs of **(A)** top views and **(B)** cross-sectional views of PEDOT-CNF composites on flexible gold electrodes with different surface charge densities ranging from 1 to 8 nC/µm^2^. PEDOT-CNF films were galvanostatically deposited on gold microelectrodes with a constant current density of 10 pA/μm^2^, using two-electrode configuration. Plots describing the evolution of deposition height **(C)** and diameter **(D)** of the composite versus deposition charge density. The error bars represent the standard deviation where *n* = 6.

The stock solution of oxidized CNFs was first vortexed for 10 min and homogenized by sonication. Next, the sample was dispersed in DI water, at a concentration of 1 mg/ml, along with 10 mM EDOT (Sigma Aldrich). Later, the CNFs-EDOT mixed suspension was incubated under vortex for 2 days at room temperature. Prior to use, the suspension containing CNFs and EDOT polymer was sonicated for 2 min and vortexed again for 15 min to obtain a homogenized dispersion. The gold microelectrodes were electrochemically cleaned by cycling at 200 mV/s between -0.3 and 1.4 V vs. Ag/AgCl in 0.5 M H_2_SO_4_ using cyclic voltammetry (Supplementary Figure S1A). PEDOT-CNF composites were galvanostatically deposited on the clean Au electrode surface by applying the current density of 10 pA/µm^2^ with a range of charge densities (1, 2, 4, 6 and 8 nC/µm^2^) (Figure 2 and Supplementary Figure S1B).

### 2.4. Optical Characterization

**FIGURE 3:**
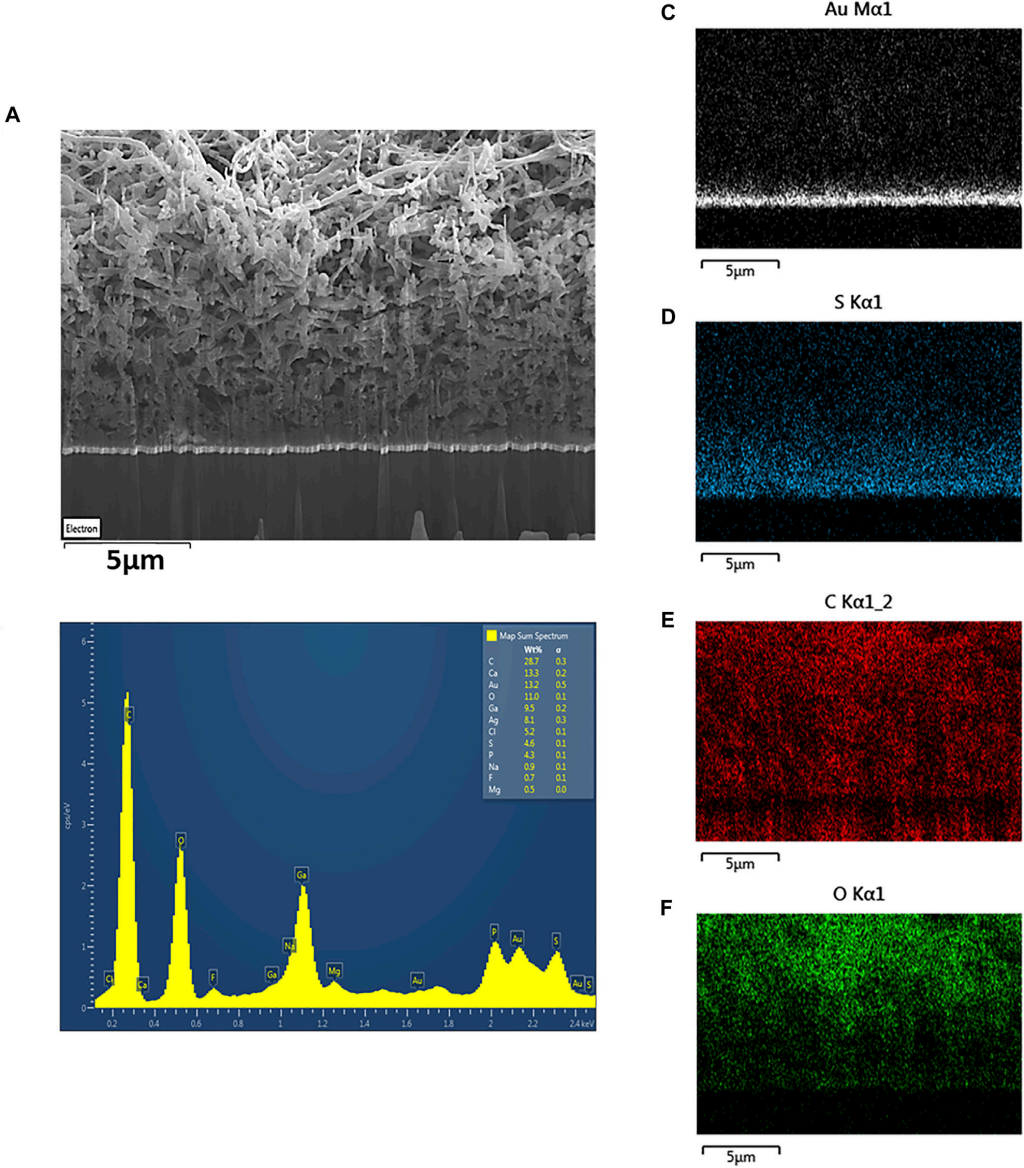
**(A)** SEM image and **(B)** EDX spectrum of porous PEDOT-CNF composite (8 nC/µm^2^) deposited on flexible gold electrodes. Elemental mapping images confirm the presence of **(C)** gold, **(D)** sulfur, **(E)** carbon and **(F)** oxygen in the PEDOT-CNF composite.

Optical microscope images of the implant, before and after the composite deposition (Figures 1D,E and Figure 7B) were obtained with a HIROX microscope (HI-SCOPE Advanced KH-3000). The morphological information of the PEDOT-CNF composite coatings on flexible microelectrode array (Figure 2) and the corresponding EDX characterization (Figure 3) were obtained by using FEG Schottky High resolution Helios 600i Dual FIB scanning electron microscope.

### 2.5. Electrochemical Characterization

**FIGURE 4:**
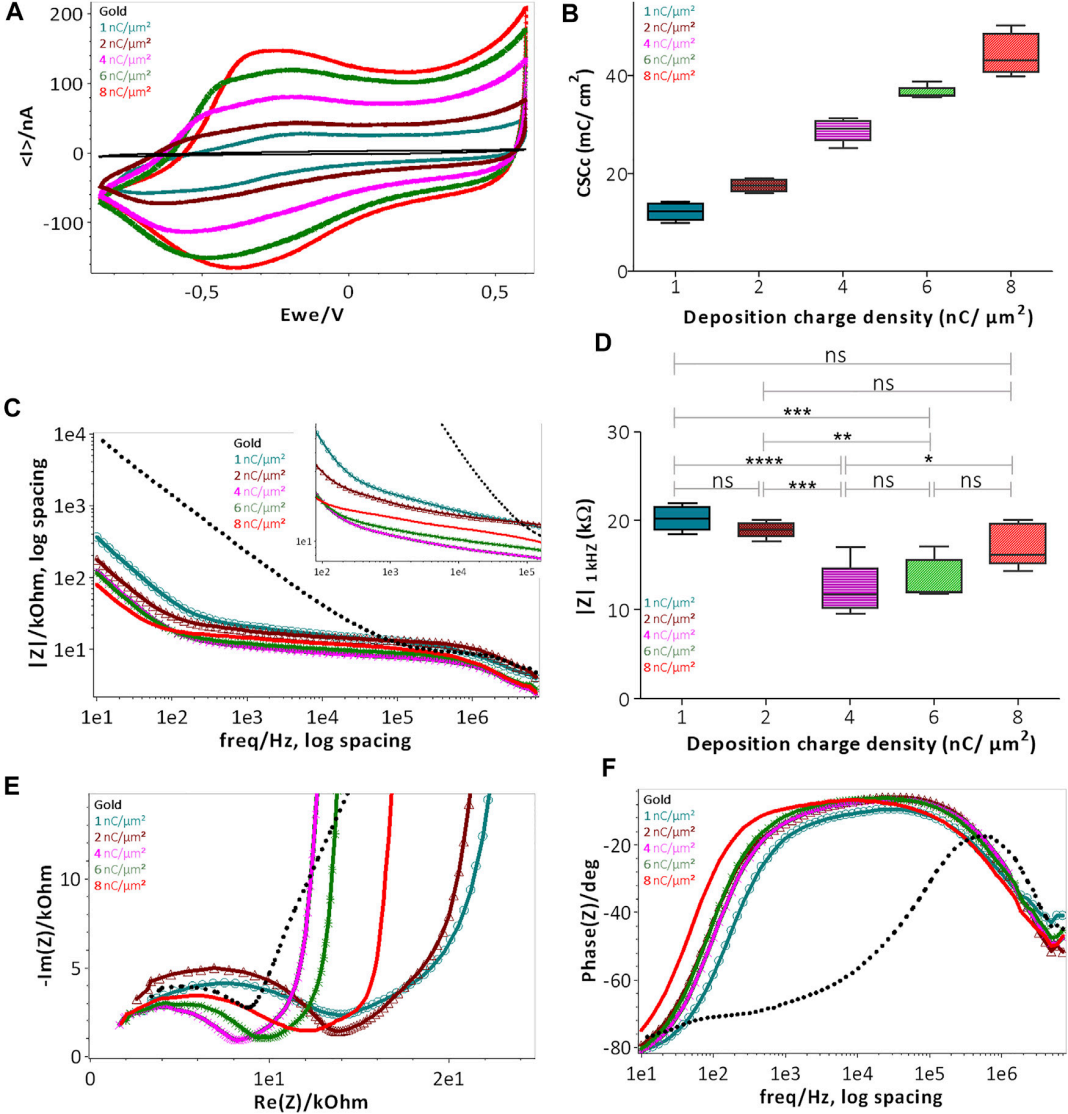
Electrochemical characterization of PEDOT-CNF composite deposited on flexible gold surface with different surface charge densities: **(A)** CSCc measurements by CV in aCSF at 200 mV/s vs. Ag/AgCl ref electrode; **(B)** Plot representing the evolution of CSCc with respect to the deposition charge density; **(C)** Bode plot representing the |Z| vs. frequency over a frequency range of 10 Hz to 7 kHz in aCSF at 0 V vs. Ag/AgCl ref electrode; **(D)** Plot of impedance |Z| _**1 kHz**_ responses vs deposition charge density. The statistical differences between deposition conditions were assessed by ANOVA followed by Tukey’s posthoc test, where ****, ***, ** and ns represent *p* < 0.0001, *p* < 0.001, *p* < 0.01 and no significant difference, respectively (*n* = 5); **(E)** Nyquist plots and **(F)** phase angle measurements obtained by EIS. Deposition charge density conditions were represented in different colors, along with the plain gold electrode (black color) in the above all graphs (For interpretation of the references to colors in this figure legend, the reader is referred to the web version of this article).

**FIGURE 5:**
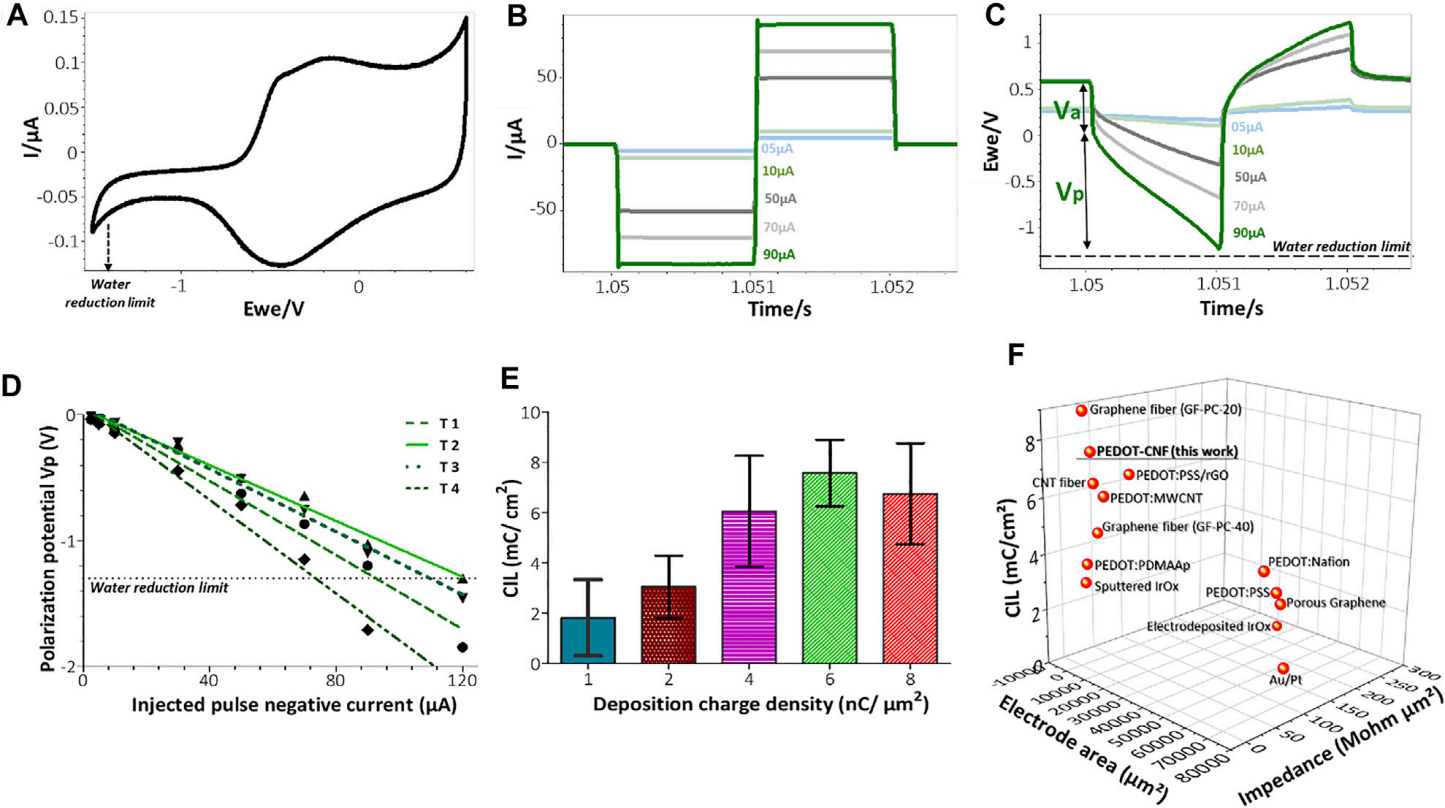
*In vitro* biphasic stimulation assessment: **(A)** Determination of the water reduction potential by CV of PEDOT-CNF composite in aCSF at 200 mV/s V Ag/AgCl. Biphasic charge-balanced current pulses **(B)** and voltage responses **(C)** at different charge injections ranging from 5 to 90 µA; **(D)** Polarization potentials (Vp) measured under different current pulse amplitudes. The deposition condition of 6 nC/µm^2^ was considered here and the data corresponding to 4 trials are represented; **(E)** Evolution of the charge injection limit (CIL) values with respect to the deposition charge density. **(F)** Comparison of the Impedance, CIL performances and geometric surface area of PEDOT-CNF composite deposited on the flexible gold electrode surface with other flexible neural electrode arrays. The corresponding values and references are reported in Supplementary Table S1 (For interpretation of the references to colors in this figure legend, the reader is referred to the web version of this article).

**FIGURE 6:**
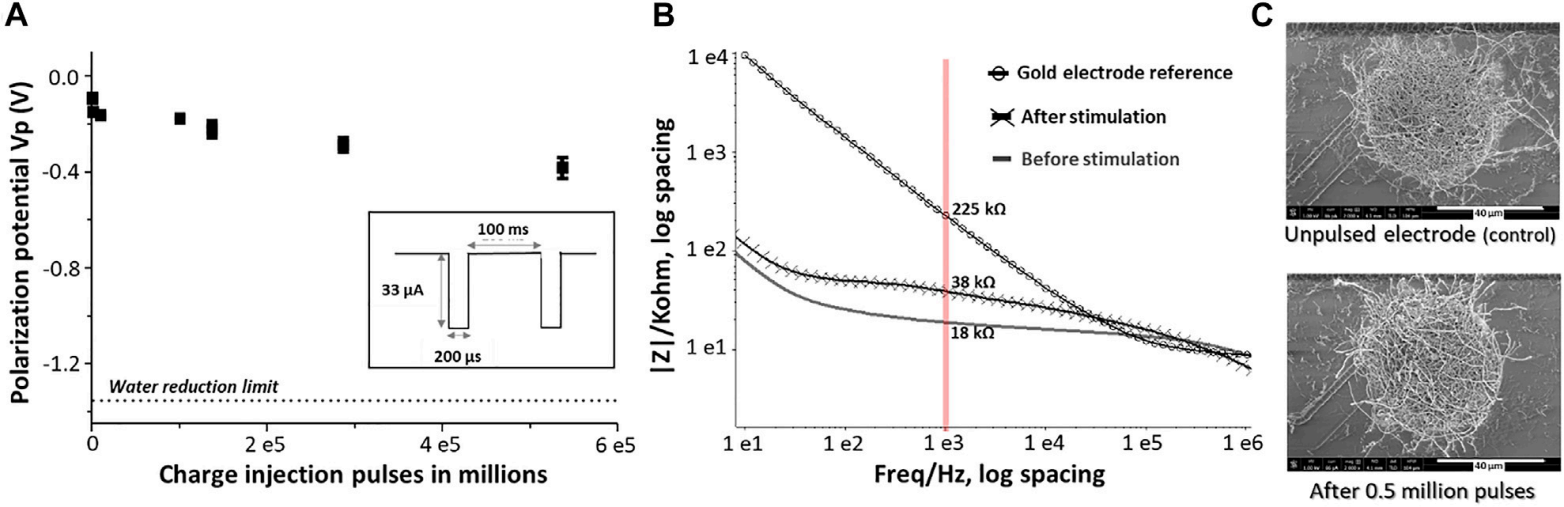
Long term stability assessment of PEDOT-CNF composite: **(A)** Evolution of polarization potential over 0.5 million charge injection pulses at 0.5 mC/cm^2^ in aCSF. Inset- Schematic representation of the biphasic charge-balanced current waveform having an amplitude of 33 µA and 200 µs pulse duration at 10 Hz. **(B)** EIS monitoring of PEDOT-CNF composite before and after 0.5 million stimulation pulses. **(C)** SEM observation of the composite after the stimulation and comparison with the unpulsed electrode (control).

Prior to the characterization, the probes were rinsed with DI water and immersed in artificial cerebrospinal fluid (aCSF) for at least 30 min. For physiological relevance, the aCSF composition mimicked the mammalian ionic CSF composition and consisted of (in mM): NaCl 124, NaHCO_3_ 26, KCl 3.2, MgSO_4_ 1, NaH_2_PO_4_ 0.5, CaCl_2_ 1.1, and glucose 10, and bubbled with 95% O_2_ and 5% CO_2_ (pH 7.4) (Gleizes et al., 2017). Probes were proceeded further with charge storage capacity (CSC) (Figures 4A,B), electrochemical impedance spectroscopy (EIS) (Figures 4C–F) and charge injection limit (CIL) measurements (Figure 5 and Figure 6). Electrochemical characterizations were performed in a three-electrode configuration, using a thick (2-mm diameter, ~5 mm^2^) Pt wire (WPI, 99.99%) as counter electrode (CE) and an Ag/AgCl reference coil electrode.

Impedance measurements (EIS) were performed between 10 Hz and 7 MHz, using a 10 mV AC signal at 0 V vs. Ag/AgCl, whereas the cathodic charge storage capacity measurements (CSCc) were carried out by launching cyclic voltammetry on a low current potentiostat channel (BioLogic VMP3), between 0.6 V and −0.8 V in aCSF at room temperature. Each electrode sample was swept for two cycles and the CSCc was calculated as the time integral of the cathodic current recorded over a potential range of 0.6 V to −0.85 V in the second cycle.

To estimate the charge injection limit, voltage transient measurements were carried out at different input currents by applying charge-balanced biphasic current pulse waveforms at 10 Hz, with pulse durations of 1 ms (Figure 5) and 200 µs (Figure 6), using a Bio-Logic VSP3 potentiostat. The negative polarization potential (V_p_) was calculated by subtracting the initial access voltage (V_a_) due to solution resistance from the total voltage (V_max_). The charge injection limits were calculated by multiplying the current amplitude and pulse duration at which the polarization potential reaches the water reduction limit (−1.3 V), divided by the geometric surface area of the electrode(Boehler et al., 2020).

Long-term stimulation stability testing was assessed by launching at least 0.5 million continuous current pulses of 33 µA at 10 Hz, thereby employing the charge injection capacity of 0.5 mC/cm^2^. The electrochemical differences before and after pulsing were measured with EIS. The electrochemical cell was sealed properly to avoid evaporation of the electrolyte during the measurements.

Accelerated aging of flexible probes was accomplished by immersion in 1x PBS at 57°C (Castagnola et al., 2015). At this temperature, a simulated 3 months (92 days) of aging *in vivo* is completed in 23 days. EIS measurements of each electrode were taken at regular intervals at 37°C.

### 2.6. Brain Slice Preparation and Electrophysiological Measurements

All procedures were conducted in accordance with the guidelines from the French Ministry of Agriculture (décret 87/848) and from the European Community (directive 86/609) and was approved by the Ministère de l’Enseignement Supérieur, de la Recherche et de l’Innovation (N° 15226-2018052417151228). Adult (>2-month-old) wild type female mice were anesthetized with isoflurane and killed by decapitation. All following procedures were made in the presence of oxygenated (95% O_2_ and 5% CO_2_) and ice-cold modified, artificial cerebrospinal fluid (maCSF) whose composition was (in mM): NaCl 124, NaHCO_3_ 26, KCl 3.2, MgSO_4_ 1, MgCl_2_ 9, NaH_2_PO_4_ 0.5, and glucose 10 (Gleizes et al., 2017). The upper part of the skull was drilled off and the whole brain was carefully removed and glued on a pedestal for slicing. 400 µm-thick coronal brain slices were cut on a vibratome (752 M vibroslice, Campden Instrument, United Kingdom), whose chamber was filled with ice-cold oxygenated maCSF. The slices were kept at room temperature for at least 1 hour in an *in vivo*-like artificial cerebrospinal fluid (aCSF, composition in “electrochemical characterization” above), aerated with 95% O_2_ and 5% CO_2_ (pH 7.4). For recording and stimulation, a brain slice was fixed on the mesh of a submersion type recording chamber (Scientific System Design, Mississauga, Ontario, Canada), as shown in the Figure 8A. The recording chamber was continuously supplied in oxygenated aCSF that was gravity fed at a flow rate of 3–3.5 ml/min. The temperature was maintained at 33–34°C. The neural microelectrodes were positioned in the hippocampal regions (CA1 and CA3) of the brain slice, using a 3D micromanipulator. Tungsten-in-epoxy lite microelectrodes (FHC, 0.2–0.3 MΩ) were also used for parallel recording and stimulation. Signals were amplified (final gain: × 10^4^) and filtered with a NeuroLog recording system (Digitimer Ltd., United Kingdom) and digitized with a 1401plus interface (CED systems, Cambridge, United Kingdom) with a digitization rate of 20 kHz. The signals were visualized online and analysed offline using spike2 software (CED) and custom scripts within Spike2 software.

## 3 Results and Discussion

### 3.1. Morphological Study of Electrodeposited PEDOT-CNF Composites on Flexible Implants

In this work, we used a flexible neural implant having an array of 4 gold micro-disk-electrodes (40-µm diameter), that are sandwiched between two parylene C layers (Figures 1A,B). A thick polymer backbone (23 µm) and thin passivation layers (1.3 µm) were opted for, such that there exists a balance between flexibility and improved long-term performance vs. potential insulation regulation. We used this design to directly compare PEDOT-CNF and bare gold microelectrodes properties (structure, morphology, electrochemical performances and stability). The electrochemical deposition of PEDOT-CNF composite is illustrated in Figures 1C–E. Before the electrodeposition, oxidized CNFs were synthesized for the following electrochemical synthesis of PEDOT-CNF composite (Rasheed et al., 2007; Bortolamiol et al., 2014; Saunier et al., 2020b). Chemical oxidation of the CNFs leads to the formation of negatively-charged functional groups *i.e.* carboxylate and hydroxyl on the outer surface of the CNFs, which render them usable for charge-balancing anionic PEDOT dopant. Later, the PEDOT-CNF nanocomposites were deposited, on the flexible implantable electrode array, where PEDOT was galvanostatically deposited along with the entrapped oxidized CNFs within its matrix in one step (Figures 1C–E). The deposition took place through a simultaneous oxidative PEDOT polymer chain propagation and CNF trapping mechanisms, resulting in a fibrous network of oxidized CNFs surrounded by PEDOT. The optimal deposition conditions were investigated by varying the surface charge densities, ranging from 1 nC/µm^2^ to 8 nC/µm^2^, at a constant current density of 10 pA/µm^2^.

SEM observations and FIB cross-sectional characterization of PEDOT-CNF composites of all deposition conditions (1–8 nC/µm^2^), showed that the entrapped CNFs were spatially distributed all over the gold electrode surface, and constitute a network of inter-connected nanofibers with variations in their aspect ratios and deposition densities (Figures 2A,B). Since the CNFs core dictates the electrochemical growth of PEDOT, a porous and fibrous structure was a common feature among all the deposits. The deposition thickness and diameter, therefore the effective surface area, increased linearly with respect to the applied charge density (Figures 2C,D). At the lowest charge densities (1 nC/µm^2^), the gold electrode surface was covered by a non-uniform layer of PEDOT-CNFs. In contrast, at higher charge deposition densities, the composite film was more uniformly porous and fibrous, thereby at the same time facilitating seamless intra- and interlayer ionic/electronic transport.

The energy dispersive X-ray (EDX) mapping data (Figure 3) illustrates the spatial distribution of gold (white), sulfur (blue), carbon (red) and oxygen (green) within the gold-PEDOT-CNF composite electrode at a surface charge deposition density of 8 nC/µm^2^. Within the composite, the sulfur reflects the presence of PEDOT and the oxygen represents both PEDOT and COOH groups on oxidized CNFs. According to Figure 3E, PEDOT is found to be densely concentrated at the gold interface, suggesting that there exists a highly ordered and less porous PEDOT-CNF composite at the gold-composite interface. In addition, the presence of sulfur was observed mostly around the walls of CNFs (Saunier et al., 2020b), indicating that PEDOT is grafted around the walls of nanofibers. Overall SEM and EDX observations suggest that PEDOT is acting as a polymer chain template to trap the oxidized CNFs and propagate all around the gold electrode surface resulting a three-dimensional growth of PEDOT around the oxidized CNFs.

### 3.2. Electrochemical Characterization (CSCc, EIS and CIL)

The electrodeposited PEDOT-CNF composite films at different deposition charge densities were electrochemically characterized *in vitro* to assess their bidirectional transduction (electrolyte/electrode) capabilities. In this regard, electrochemical impedance spectroscopy (EIS), cathodic charge storage capacity (CSCc) and charge injection limits (CIL) are the essential parameters. On the one hand, a minimal impedance value is required to achieve signal noise reduction, such as thermal noise through shunt pathways (Cogan 2008; Ghane-Motlagh and Sawan 2013; Viswam et al., 2019). On the other hand, large charge storage capacity and maximized charge injection limit values are particularly desired to establish safe electrical stimulation (Cogan 2008; Ghane-Motlagh and Sawan 2013).

#### 3.2.1. CSCc and EIS Measurements

The evaluation of the charge transfer capabilities of PEDOT-CNF composites was carried out in aCSF, a physiologically relevant media, by sweeping a potential range between −0.85 and 0.6 V, at a scan rate of 200 mV/s, using cyclic voltammetry (CV). This technique provides insights regarding the electrode interface under electrical load, the electrochemical conversion of species within the solution, and the transient changes due to redox reactions at the electrode surface (Cogan 2008). The cathodal CSC (CSCc) of the composite film was calculated as the time integral of the cathodal currents within the cycled region. As indicated in Figures 4A,B, the composite deposition on the gold microelectrode resulted in a progressive increment in the average CSCc values, up to 48 ± 4 mC/cm^2^, much higher than that of the bare gold electrode (1 ± 0.35 mC/cm^2^). There is a linear relationship between the charge delivered to the electrode during the deposition and charge storage capacity (Figure 4B). This behavior is likely due to the surface area increment that allows for the effective diffusion of electrolyte ions at the electrode-solution interface.

The EIS magnitude and phase angle measurements of the composite electrodes were measured over a range of frequencies from 10 Hz to 7 MHz (Figures 4C–F). The phase angle measurements of the PEDOT-CNF modified electrodes at lower frequency range (~10 Hz), revealed that the capacitive behavior was predominant with an angle around 80° (Figure 4F). The angle shift towards resistive charge transfer, for a frequency range of 10 Hz to 10 kHz was proportional to the deposition charge density *i.e.*, the higher the effective surface area, the larger the phase angle shift (Kozai et al., 2016; Zhou et al., 2013). Figure 4C represents the bode plot displaying the impedance magnitude (|Z|) *vs.* frequency. PEDOT-CNF modified electrodes show an impedance range of 10–20 kΩ, which is at least 10 times less than that of the bare gold electrode (200–400 kΩ). Impedance values of the 5 deposition conditions were analyzed using one-way ANOVA followed by post-hoc Turkey’s test (*n* = 5). ANOVA evidenced a significant effect of deposition charge density on |Z|_1 kHZ_ (*p* < 0.0001), yet changes in |Z|_1 kHZ_ were not proportional to the charge density, where the values at |Z|_1 kHZ_ being the most commonly used characteristic frequency band for action potentials (Boehler et al., 2020). In comparison to the 1 nC/µm^2^ charge density, significant lowering of |Z|_1 kHZ_ was only obtained with charge densities of 4 nC/µm^2^ and 6 nC/µm^2^ (*p* < 0.0001 and *p* = 0.0006 respectively, Tukey’s test). |Z|_1 kHZ_ obtained with charge densities of 2 and 8 nC/µm^2^ did not differ from that at 1 nC/µm^2^ (*p* = 0.3 and 0.06 respectively). The conditions 4 nC/µm^2^ and 6 nC/µm^2^ showed no statistically significant differences (*p* = 0.09). However, the optimal deposition condition seemed to be that at 6 nC/µm^2^ as it displayed both a sharp impedance decrement on average and the smallest variability across electrodes, with the |Z|_1 kHz_ value being 13.4 ± 2.2 kΩ (16.8 ± 2 MΩ µm^2^).

To follow up further the investigation of the influence of the electrode surface area on the electrode performance, Nyquist plots were made to monitor the evolution of charge transfer resistance (semi-circle region) as a function of deposition charge on the electrode. As illustrated in Figure 4E the diameter of the semi-circle region becomes smaller with the increase in deposition charge, where it is governed by the electrode thickness, porosity and integrity of the Au-PEDOT-CNF composite interface (Gruet et al., 2019; Huang et al., 2020). Among all the deposition conditions, 4 nC/µm^2^ and 6 nC/µm^2^ clearly showed depressed semi-circle regions, thus reflecting the improved area and highly conductive surface of the electrodes with lower impedance, possessing the capability of seamless bidirectional transfer of charges/electrons.

In the 8 nC/µm^2^ charge deposition case, even though the thickness and porosity of the electrode improved the effective surface area, it also probably induced mechanical stress at the Au-PEDOT-CNF interface, resulting in a slightly increased semi-circle region, therefore a charge transfer resistance and an impedance with larger variance compared to the 6 nC/µm^2^ condition. Overall, our results indicate that a charge density of 6 nC/µm^2^ optimized the deposition of PEDOT-CNF composite on the flexible gold electrode surface resulting in a specific impedance value of 16.8 ± 2 MΩ µm^2^ at 1 kHz. This value is on par with the high-performance CNT fiber (20.5 MΩ µm^2^) (Vitale et al., 2015) and graphene fiber (9–28 MΩ µm^2^) (Wang et al., 2019) microelectrodes, and 2 to 7 times lower, than most of the electrode materials deposited on flexible metallic substrates reported in the literature (Figure 5F). The corresponding values and references are reported in Supplementary Table S1.

#### 3.2.2. Electrical Stimulation

As shown in the Figure 4A, CSCc measurements with respect to the deposition charge density were used to identify the amount of charge available in the cathodic region of their respective CV sweep. Although CV at slow scan rate provides information related to the electrochemical reactions that occur at the electrode/electrolyte interface, it cannot reflect the amount of charge available during sub-millisecond stimulation pulses. Charge balanced square wave current pulses are generally used in electrical stimulation for electrophysiology experiments and therapies, with pulse widths ranging from 50 to 1,000 µs. A high CIL would be beneficial for these extremely fast charge-discharge processes by preventing damages to the tissue-electrode region by reducing the incidence of irreversible Faradic reactions (Cogan 2008; Boehler et al., 2020). To assess the charge stimulation capability of the PEDOT-CNF composite, as a prerequisite, identifying the water electrolysis limits, especially the water reduction limit in physiologically relevant solution is important. Figure 5A shows that the water reduction voltage of PEDOT-CNF modified gold electrodes is around −1.3 V. Next, the voltage excursions in response to biphasic, cathodic first, current pulses were recorded with a 1,000 µs pulse width in aCSF (Figures 5B–D). Using a range of pulse current intensities, we defined the CIL as the amount of charge injected which caused polarization (V_p_) of the electrode beyond its water hydrolysis window. From the lowest to highest deposition charge density, the CIL values increased linearly up to the condition 6 nC/µm^2^, being the best one among all, with an average value of 7.6 ± 1.3 mC/cm^2^ (Figure 5E). This result agreed with the corresponding impedance measurements where the 6 nC/µm^2^ deposition condition provided a low impedance value with lowest variability (Figure 4D).

The optimized CIL value from this work was compared with other PEDOT based composites (Carli et al., 2019; Kleber et al., 2019; Lee et al., 2019) and porous metals deposits (Wang et al., 2009; Vitale et al., 2015; Lu et al., 2016; Shin et al., 2016; Nimbalkar et al., 2018) on flexible neural implants. Figure 5F and Supplementary Table S1 shows that, among all reported electrode materials deposited on flexible metallic substrates, the PEDOT-CNF coating displayed superior electrical properties and charge injection capabilities, thanks to the chemical composition of the material and its physical morphology.

#### 3.2.3. Long Time Performance

Along with the charge injection capability, it was also important to assess the biphasic charge cycling endurance. For this purpose, the electrodes were subjected to a series of charge balanced current pulses in physiologically relevant aCSF. Our electrodes were repeatedly pulsed over 0.5 million times using the 200 µs cathodic pulse width, followed by an immediate charge-compensating anodic pulse (most commonly reported) (Boehler et al., 2020), consisting in a charge injection density of 0.5 mC/cm^2^ (current amplitude of 33 µA). Figure 6A shows the evolution of the polarization potential (V_p_) during the time of charge injection pulsing. A resultant V_p_ drift of 0.3 V, from −0.1 V to −0.4 V, was observed after 0.5 million pulses, but remained far from the water window critical limit (−1.3 V) throughout the stimulation. The corresponding |Z|_1 kHz_ value was increased from an initial value of 18 kΩ to a final value of 38 kΩ after 0.5 million biphasic stimulation cycles, while still being far from the bare non-coated Au electrode |Z|_1 kHz_ value (>200 kΩ) (Figure 6B). In addition, the SEM images of the composite after the stimulation *vs* control, from the Figure 6C, suggested that the electrodes exhibited no significant physical delamination or degradation even after 0.5 million biphasic stimulation cycles confirming an excellent structural control and longevity.

**FIGURE 7:**
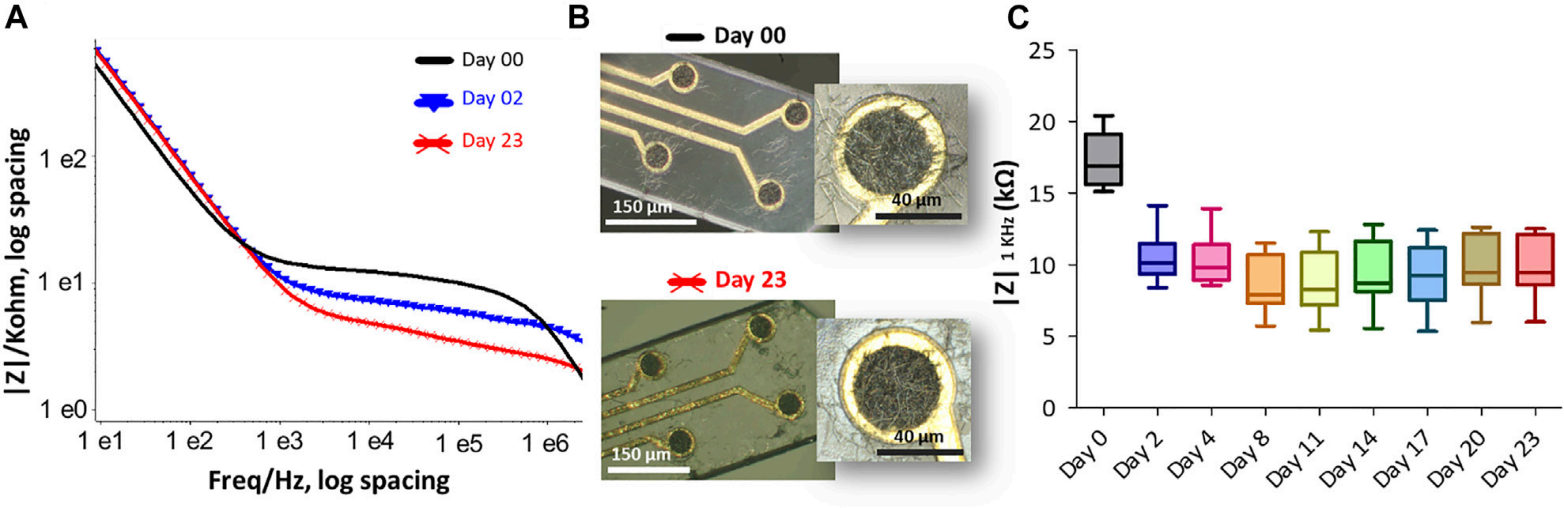
The effect of accelerated thermal aging on the PEDOT-CNF composite was examined in PBS buffer at 57°C for 23 days. **(A)** Impedance magnitude of PEDOT-CNF composite at Day 0 (black), Day 2 (blue) and Day 23 (red). Platinum wire was used as counter and reference electrodes. **(B)** Hirox microscope images of flexible probe (black) before immersion and (red) after 23 days of soaking in PBS at 57°C. **(C)** The evolution of electrode impedance at 1 KHz represented in a standard box-and-whisker plot where the sample number *n* = 8.

Accelerated thermal ageing tests were also carried out to evaluate the structural and electrical changes of the PEDOT-CNF coating as a function of time which can affect an electrode in a permanent deep brain implantation. Here, we maintained the material in PBS buffer for 23 days at 57°C, that is considered to be equivalent to an ageing of 92 days at 37°C (Castagnola et al., 2015). Figure.7A shows the Bode graph of the impedance before and after the ageing of the PEDOT-CNF microelectrodes on the flexible parylene probe, with the corresponding evolution of the impedance |Z| _1 kHz_ responses vs. time (Figure 7C). After 23 days of ageing at 57°C, the PEDOT-CNF microelectrodes (|Z| _1 kHz_ = 9.9 ± 2.2) is quite similar to the PEDOT- CNF microelectrode before ageing (|Z| _1 kHz_ = 17.3 ± 1.9). From the optical images, we observed that thermal ageing process does not impact the morphology of the PEDOT-CNF deposition (Figure 7B). This ageing tests allow us to conclude about the good mechanical and electrical stability of the PEDOT- CNF material deposited on a flexible gold electrode surface.

### 3.3. Brain Slice Electrophysiological Recording and Stimulation

**FIGURE 8:**
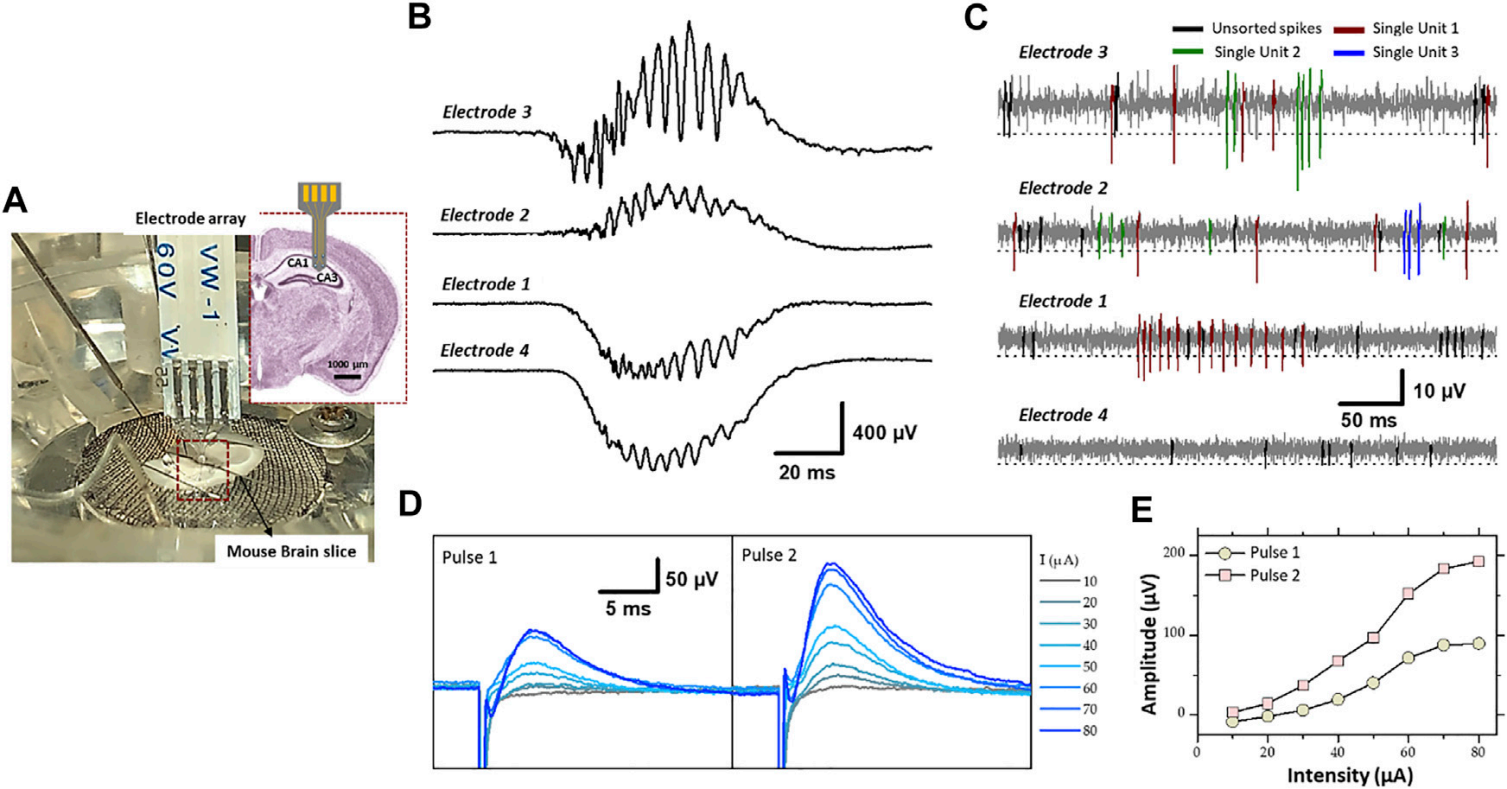
**(A)** Experimental set-up of the electrophysiological recording and stimulation, in the hippocampal region (CA1 and CA3) of a mouse brain slice, using flexible microelectrode array modified with PEDOT-CNF composite. Recording of spontaneous **(B)** sharp wave-ripples (filter: 0.1 Hz–3 kHz) and **(C)** firing of action potentials from neurons (filter: 300–3,000 Hz). Electrodes 3 and 2 were located in the pyramidal cell layer of CA1, and electrode 1 and 4 were in the dendrite layers. A threshold of -3xRMS [dashed line in **(C)**] was used for spike sorting. Single-units were identified using PCA and cluster analysis. Single-units are differentiated by color codes. Spikes in black correspond to unsorted (multiunit) activity. The traces in **(B,C)** were not obtained simultaneously but sequentially. **(D)** Evoked field potential recorded in the pyramidal cell layer after electrical stimulation of the Schaffer collateral. Pairs of cathodic square current pulses (200 µs width), with an interpulse interval of 20 ms, were applied at 0.5 Hz. Responses evoked by the first and second stimulus of the pairs are shown separately (pulse 1 and pulse 2). The different traces correspond to the response obtained at different stimulation intensities (10–80 µA). Each trace is the average of 10–20 responses obtained at a given intensity. **(E)** Field potential amplitude represented as a function of stimulation intensity (For interpretation of the references to colours in this figure legend, the reader is referred to the Web version of this article).

Electrophysiological experiments were conducted to assess the recording quality of PEDOT-CNF electrodes and their usability as stimulating electrodes (Figure 8A). Electrical stimulation and recordings were performed in the hippocampus (CA1 and CA3 regions) of mouse brain slice maintained *in vitro* (Buzsáki 2015). PEDOT-CNF modified electrodes allowed to record two types of spontaneous neuronal activities: sharp wave-ripples (SWR) complexes and neuronal spiking activity.

SWR complexes constitute a mesoscopic signal that reflects synchronized activity in large population of neurons (Buzsáki 2015). SWR complexes were recorded with minimal filtering (0.1 Hz- 3 KHz) where two electrodes of the flexible four electrode probe were located in the cell layer of CA1 and other two in the dendrite layer (stratum radiatum/lacumosum). As shown in the Figure 8B, the slow component of the SWR complex, “the sharp wave” proper, shows an inversion in polarity between the two regions: positive in the cell layer (electrodes 3 and 2) and negative in the dendrite layer (electrodes 1 and 4), as expected given the fact that the excitatory synaptic inputs, which generate the sharp wave are located on the dendrite of hippocampal pyramidal cells. The ripples correspond to the oscillatory pattern (200–250 Hz) carried by the sharp wave. The amplitude of ripples was larger in the cell body layer (electrode 3 and 2), as expected since they mostly correspond to the population spikes patterned by the local inhibitory neurons.

The other type of spontaneous activity that was recorded in the hippocampus corresponds to action potentials APs (spikes) generated by neurons (Buzsáki 2015; Obien et al., 2015). They were visualized between sharp wave-ripple complexes while using bandpass filtering (300–3,000 Hz). The examples presented in Figure 8C, were obtained with the same PEDOT-CNF modified electrodes and at the same electrode locations as in Figure 8B. The APs are recognized as fast and mostly negative deflections of varying amplitude. To proceed further we examined whether single-unit activity, i.e., APs that can be attributed to one single neuron, could be extracted from the raw traces. First portions of trace around events were extracted using a threshold at 3xRMS of the voltage trace (dashed lines in Figure 8C), and thereafter analyzed by PCA and clustering (not illustrated). This analysis allowed identifying constant spike shapes, which were further ascribed to single-unit activities if the inter-spike interval distribution (not illustrated) showed a clear refractory period, i.e., no interval <1 ms. In contrast, multiunit activities (black spikes in Figure 8C) do not have a refractory period because they are produced by several independent neurons. As shown in Figure 8C, two and three single-units (color coded) have been isolated from electrodes 3 and 2, respectively and only one single-unit was isolated with electrode 1. This is likely due to the fact that only few neuronal cell bodies, from which action potentials are generated, are to be found in the dendrite layers of the hippocampus. In summary, the resulting signal-to-noise ratio of the PEDOT-CNF electrodes facilitated the isolation of single-unit activity from neighboring neurons.

For comparison, the signal obtained with the non-coated bare gold electrodes is mainly composed of noise, with an amplitude comparable to that usually obtained before inserting the probe in the brain (Supplementary Figure S2). This is a consequence of the small diameter of the bare electrode active area (40 μm), which resulted in a high impedance. For neuronal recording, the electrode impedance contributes to the noise, and high impedance electrodes degrades the signal to noise ratio (SNR) through a combined effect of higher noise levels. On the opposite, lowering the impedance of the same electrodes with PEDOT-CNF coatings (Figure 4C) enabled the recording of very adequate electrophysiological signals with a good SNR. From the results, the SNR (±SD) was calculated as the amplitude of the negative component of the action potential of the isolated single unit divided by the RMS. The results show that the SNR is high enough (5.3 ± 1.2) (*n* = 6) for proper action potentials APs (spikes) recordings.

In addition, the PEDOT-CNF electrodes were validated for their usability as stimulating electrodes. Electrical stimulation was applied through a PEDOT-CNF electrode positioned in the dendritic layer (stratum radiatum) to activate axons of the Schaffer collateral (the projection issued from CA3 pyramidal cells and synapsing on the dendrites of CA1 pyramidal cells) (Figures 8D,E). The extracellular recording of the local field potential was made in the cell body layer of CA1 with a conventional metallic electrode. Electrical stimulus were given in pairs (20 ms interpulse interval) and, consisted in cathodic square current pulses (200 µs) delivered at different intensities. Pulse pairs were repeated at a frequency of 0.5 Hz to examine short-term plasticity of the synaptic responses. For suprathreshold intensities, the potential was positive due to recording in the cell layer (current sink in the dendrites), and the amplitude of the response to the second pulse of the pair was larger than that of the first one, reflecting short-term facilitation of the synaptic response (Figure 8E). The amplitude of the potential increased in proportion to the stimulation intensity, with a saturation at around 80 µA (Figure 8C). This saturation suggests that all the axons implicated in the response were recruited with an intensity ≤80 µA. Thus, PEDOT-CNF microelectrodes could be used as efficient stimulating electrodes, to obtain typical evoked potentials with charge densities that remained below the water reduction limit.

## 4. Conclusion

We have developed a well-controlled and versatile surface modification method for preparing macroporous PEDOT-CNFs microelectrodes on flexible implantable neural probes. We investigated by FIB and EDX the mechanism by which a macroporous nanostructure of PEDOT-CNF layer was created, where the conducting PEDOT polymer was covered uniformly and tightly around the oxidized carbon nanofibers as a solid doping template. We found that the combination of carbon nanofibers and PEDOT as a single carbonaceous composite resulted in a strong synergetic effect leading to a lower impedance, superior charge storage capacity and charge injection limit compared to bare metal and other reported organic coated flexible electrodes in electrochemical characterization. We further showed that the carbon nanofibers function as reinforcing elements within the composite material and prevent PEDOT film from undergoing delamination or cracking during long lasting electrical pulsing experiments. *In vitro* experiments on mouse brain slices showed that the PEDOT-CNFs microelectrodes can record spontaneous oscillatory field potentials as well as single-unit action potentials with good signal-to-noise ratio, and allow to safely deliver electrical stimulation for evoking field potentials. These results show that these electrodes are well suited for high-performance recording and/or stimulation for applications in brain therapies.

Despite the potential of the PEDOT-CNFs composites that can bring, the non-toxicity properties of these nanofibrous materials in creating neural interfaces, need to be fully studied. Our preliminary *in vitro* cell viability experiments (MTT cell viability assay ISO 10993-5 norm) on PEDOT-CNFs coated microelectrodes showed that the nanocomposite material does not advocate any cytotoxicity, neither detachment of the CNFs from the bare electrodes (Saunier et al., 2020b). The results showed that the population of living cells increased over time in a similar fashion for controls, demonstrating that no cytotoxicity could be observed “*in vitro*” as viability percentage highly exceeds 75%. However, precise toxicity effects of these nanofibers when implanted in the brain need further investigation. Importantly, the risk that may arise from detaching CNF during the insertion procedure should be considered. The physical dimension characteristics is a key factor promoting the risk of toxicity to biological systems and should not be ignored. Recent studies on the effect of various carbon nanofibers and nanotubes on human epithelial cells, showed association between the physical dimensions and genotoxicity, especially those with greater lengths and larger diameters (Fraser et al., 2020). In our future works, we envision to examine the effects of the PEDTOT-CNFs through immunohistochemistry in animal models, followed by comparison of the results against existing non-coated electrodes.

## Data Availability

The original contributions presented in the study are included in the article/Supplementary Material, further inquiries can be directed to the corresponding author.

## References

[B1] Abidian M. R. Ludwig K. A. Marzullo T. C. Martin D. C. Kipke D. R. (2009). Interfacing Conducting Polymer Nanotubes with the Central Nervous System: Chronic Neural Recording Using Poly(3,4-Ethylenedioxythiophene) Nanotubes. Adv. Mater. 21 (37), 3764–3770. 10.1002/adma.200900887 26345408 PMC4559350

[B2] Alba N. Du Z. Catt K. Kozai T. Cui X. (2015). *In Vivo* electrochemical Analysis of a PEDOT/MWCNT Neural Electrode Coating. Biosensors 5 (4), 618–646. 10.3390/bios5040618 26473938 PMC4697137

[B3] Ansaldo A. Castagnola E. Maggiolini E. Fadiga L. Ricci D. (2011). Superior Electrochemical Performance of Carbon Nanotubes Directly Grown on Sharp Microelectrodes. ACS nano 5 (3), 2206–2214. 10.1021/nn103445d 21341752

[B4] Bareket-Keren L. Hanein Y. (2013). Carbon Nanotube-Based Multi Electrode Arrays for Neuronal Interfacing: Progress and Prospects. Front. Neural Circuits 6, 122. 10.3389/fncir.2012.00122 23316141 PMC3540767

[B5] Benabid A. L. (2003). Deep Brain Stimulation for Parkinson's Disease. Curr. Opin. Neurobiol. 13 (6), 696–706. 10.1016/j.conb.2003.11.001 14662371

[B6] Bhandari S. Deepa M. Srivastava A. K. Joshi A. G. Kant R. (2009). Poly(3,4-ethylenedioxythiophene)−Multiwalled Carbon Nanotube Composite Films: Structure-Directed Amplified Electrochromic Response and Improved Redox Activity. J. Phys. Chem. B 113 (28), 9416–9428. 10.1021/jp9012976 19545156

[B7] Boehler C. Carli S. Fadiga L. Stieglitz T. Asplund M. (2020). Tutorial: Guidelines for Standardized Performance Tests for Electrodes Intended for Neural Interfaces and Bioelectronics. Nat. Protoc. 15 (11), 3557–3578. 10.1038/s41596-020-0389-2 33077918

[B8] Boehler C. Oberueber F. Schlabach S. Stieglitz T. Asplund M. (2017). Long-term Stable Adhesion for Conducting Polymers in Biomedical Applications: IrOx and Nanostructured Platinum Solve the Chronic challenge. ACS Appl. Mater. Inter. 9 (1), 189–197. 10.1021/acsami.6b13468 27936546

[B9] Bongo M. Winther-Jensen O. Himmelberger S. Strakosas X. Ramuz M. Hama A. (2013). PEDOT:gelatin Composites Mediate Brain Endothelial Cell Adhesion. J. Mater. Chem. B 1 (31), 3860–3867. 10.1039/c3tb20374c 32261140

[B10] Bortolamiol T. Lukanov P. Galibert A.-M. Soula B. Lonchambon P. Datas L. (2014). Double-walled Carbon Nanotubes: Quantitative Purification Assessment, Balance between Purification and Degradation and Solution Filling as an Evidence of Opening. Carbon 78, 79–90. 10.1016/j.carbon.2014.06.051

[B11] Buzsáki G. (2015). Hippocampal Sharp Wave‐ripple: A Cognitive Biomarker for Episodic Memory and Planning. Hippocampus 25 (10), 1073–1188. 10.1002/hipo.22488 26135716 PMC4648295

[B12] Carli S. Bianchi M. Zucchini E. Di Lauro M. Prato M. Murgia M. (2019). Electrodeposited PEDOT:Nafion Composite for Neural Recording and Stimulation. Adv. Healthc. Mater. 8 (19), 1900765. 10.1002/adhm.201900765 31489795

[B13] Castagnola V. Descamps E. Lecestre A. Dahan L. Remaud J. Nowak L. G. (2015). Parylene-based Flexible Neural Probes with PEDOT Coated Surface for Brain Stimulation and Recording. Biosens. Bioelectron. 67, 450–457. 10.1016/j.bios.2014.09.004 25256782

[B14] Cogan S. F. (2008). Neural Stimulation and Recording Electrodes. Annu. Rev. Biomed. Eng. 10, 275–309. 10.1146/annurev.bioeng.10.061807.160518 18429704

[B15] Cui X. T. Zhou D. D. (2007). Poly (3,4-Ethylenedioxythiophene) for Chronic Neural Stimulation. IEEE Trans. Neural Syst. Rehabil. Eng. 15 (4), 502–508. 10.1109/tnsre.2007.909811 18198707

[B16] Fabretto M. V. Evans D. R. Mueller M. Zuber K. Hojati-Talemi P. Short R. D. (2012). Polymeric Material with Metal-like Conductivity for Next Generation Organic Electronic Devices. Chem. Mater. 24 (20), 3998–4003. 10.1021/cm302899v

[B17] Fraser K. Kodali V. Yanamala N. Birch M. E. Cena L. Casuccio G. (2020). Physicochemical Characterization and Genotoxicity of the Broad Class of Carbon Nanotubes and Nanofibers Used or Produced in U.S. Facilities. Part. Fibre Toxicol. 17 (1), 62–26. 10.1186/s12989-020-00392-w 33287860 PMC7720492

[B18] Gerwig R. Fuchsberger K. Schroeppel B. Link G. S. Heusel G. Kraushaar U. (2012). PEDOT-CNT Composite Microelectrodes for Recording and Electrostimulation Applications: Fabrication, Morphology, and Electrical Properties. Front. Neuroeng. 5, 8. 10.3389/fneng.2012.00008 22586394 PMC3343311

[B19] Ghane-Motlagh B. Sawan M. (2013). Design and Implementation Challenges of Microelectrode Arrays: a Review. Msa 04 (08), 483–495. 10.4236/msa.2013.48059

[B20] Gleizes M. Perrier S. P. Fonta C. Nowak L. G. (2017). Prominent Facilitation at Beta and Gamma Frequency Range Revealed with Physiological Calcium Concentration in Adult Mouse Piriform Cortex *In Vitro* . PloS one 12 (8), e0183246. 10.1371/journal.pone.0183246 28820903 PMC5562311

[B21] Green R. Abidian M. R. (2015). Conducting Polymers for Neural Prosthetic and Neural Interface Applications. Adv. Mater. 27 (46), 7620–7637. 10.1002/adma.201501810 26414302 PMC4681501

[B22] Grill W. M. Norman S. E. Bellamkonda R. V. (2009). Implanted Neural Interfaces: Biochallenges and Engineered Solutions. Annu. Rev. Biomed. Eng. 11, 1–24. 10.1146/annurev-bioeng-061008-124927 19400710

[B23] Gruet D. Delobel B. Sicsic D. Lucas I. T. Vivier V. (2019). On the Electrochemical Impedance Response of Composite Insertion Electrodes - toward a Better Understanding of Porous Electrodes. Electrochimica Acta 295, 787–800. 10.1016/j.electacta.2018.10.115

[B24] Hess L. H. Jansen M. Maybeck V. Hauf M. V. Seifert M. Stutzmann M. (2011). Graphene Transistor Arrays for Recording Action Potentials from Electrogenic Cells. Adv. Mater. 23 (43), 5045–5049. 10.1002/adma.201102990 21953832

[B25] Huang J. Gao Y. Luo J. Wang S. Li C. Chen S. (2020). Review—Impedance Response of Porous Electrodes: Theoretical Framework, Physical Models and Applications. J. Electrochem. Soc.

[B26] Inal S. Rivnay J. Suiu A.-O. Malliaras G. G. McCulloch I. (2018). Conjugated Polymers in Bioelectronics. Acc. Chem. Res. 51 (6), 1368–1376. 10.1021/acs.accounts.7b00624 29874033

[B27] Jan E. Hendricks J. L. Husaini V. Richardson-Burns S. M. Sereno A. Martin D. C. (2009). Layered Carbon Nanotube-Polyelectrolyte Electrodes Outperform Traditional Neural Interface Materials. Nano Lett. 9 (12), 4012–4018. 10.1021/nl902187z 19785391

[B28] Jeong J.-W. Shin G. Park S. I. Yu K. J. Xu L. Rogers J. A. (2015). Soft Materials in Neuroengineering for Hard Problems in Neuroscience. Neuron 86 (1), 175–186. 10.1016/j.neuron.2014.12.035 25856493

[B29] Jun J. J. Steinmetz N. A. Siegle J. H. Denman D. J. Bauza M. Barbarits B. (2017). Fully Integrated Silicon Probes for High-Density Recording of Neural Activity. Nature 551 (7679), 232–236. 10.1038/nature24636 29120427 PMC5955206

[B30] Kellis S. Sorensen L. Darvas F. Sayres C. O’Neill K. III Brown R. B. (2016). Multi-scale Analysis of Neural Activity in Humans: Implications for Micro-scale Electrocorticography. Clin. Neurophysiol. 127 (1), 591–601. 10.1016/j.clinph.2015.06.002 26138146

[B31] Khodagholy D. Doublet T. Gurfinkel M. Quilichini P. Ismailova E. Leleux P. (2011). Highly Conformable Conducting Polymer Electrodes for *In Vivo* Recordings. Adv. Mater. 23 (36), H268–H272. 10.1002/adma.201102378 21826747

[B32] Khodagholy D. Gelinas J. N. Thesen T. Doyle W. Devinsky O. Malliaras G. G. (2015). NeuroGrid: Recording Action Potentials from the Surface of the Brain. Nat. Neurosci. 18 (2), 310–315. 10.1038/nn.3905 25531570 PMC4308485

[B33] Kleber C. Lienkamp K. Rühe J. Asplund M. (2019). Wafer‐Scale Fabrication of Conducting Polymer Hydrogels for Microelectrodes and Flexible Bioelectronics. Adv. Biosys. 3 (8), 1900072. 10.1002/adbi.201900072 32648703

[B34] Kozai T. D. Y. Catt K. Du Z. Na K. Srivannavit O. Haque R.-u. M. (2016). Chronic *In Vivo* Evaluation of PEDOT/CNT for Stable Neural Recordings. IEEE Trans. Biomed. Eng. 63 (1), 111–119. 10.1109/tbme.2015.2445713 26087481 PMC4688254

[B35] Larsen S. T. Vreeland R. F. Heien M. L. Taboryski R. (2012). Characterization of Poly(3,4-Ethylenedioxythiophene):tosylate Conductive Polymer Microelectrodes for Transmitter Detection. Analyst 137 (8), 1831–1836. 10.1039/c2an16288a 22383043

[B36] Lecomte A. Degache A. Descamps E. Dahan L. Bergaud C. (2017). *In Vitro* and *In Vivo* Biostability Assessment of Chronically-Implanted Parylene C Neural Sensors. Sensors Actuators B: Chem. 251, 1001–1008. 10.1016/j.snb.2017.05.057

[B37] Lee S. Eom T. Kim M.-K. Yang S.-G. Shim B. S. (2019). Durable Soft Neural Micro-electrode Coating by an Electrochemical Synthesis of PEDOT:PSS/Graphene Oxide Composites. Electrochimica Acta 313, 79–90. 10.1016/j.electacta.2019.04.099

[B38] Lu Y. Lyu H. Richardson A. G. Lucas T. H. Kuzum D. (2016). Flexible Neural Electrode Array Based-On Porous Graphene for Cortical Microstimulation and Sensing. Sci. Rep. 6 (1), 33526–33529. 10.1038/srep33526 27642117 PMC5027596

[B39] Luo X. Weaver C. L. Tan S. Cui X. T. (2013). Pure Graphene Oxide Doped Conducting Polymer Nanocomposite for Bio-Interfacing. J. Mater. Chem. B 1 (9), 1340–1348. 10.1039/c3tb00006k 25984340 PMC4433042

[B40] Luo X. Weaver C. L. Zhou D. D. Greenberg R. Cui X. T. (2011). Highly Stable Carbon Nanotube Doped Poly(3,4-Ethylenedioxythiophene) for Chronic Neural Stimulation. Biomaterials 32 (24), 5551–5557. 10.1016/j.biomaterials.2011.04.051 21601278 PMC3109196

[B41] Mayberg H. S. Lozano A. M. Voon V. McNeely H. E. Seminowicz D. Hamani C. (2005). Deep Brain Stimulation for Treatment-Resistant Depression. Neuron 45 (5), 651–660. 10.1016/j.neuron.2005.02.014 15748841

[B42] Maziz A. Concas A. Khaldi A. Stålhand J. Persson N. K. Jager E. W. (2017). Knitting and Weaving Artificial Muscles. Sci. Adv. 3 (1), e1600327. 10.1126/sciadv.1600327 28138542 PMC5266480

[B43] Maziz A. Özgür E. Bergaud C. Uzun L. (2021). Progress in Conducting Polymers for Biointerfacing and Biorecognition Applications. Sensors Actuators Rep. 3, 100035. 10.1016/j.snr.2021.100035

[B44] Maziz A. Plesse C. Soyer C. Cattan E. Vidal F. (2016). Top-down Approach for the Direct Synthesis, Patterning, and Operation of Artificial Micromuscles on Flexible Substrates. ACS Appl. Mater. Inter. 8 (3), 1559–1564. 10.1021/acsami.5b09577 26709595

[B45] Maziz A. Plesse C. Soyer C. Chevrot C. Teyssié D. Cattan E. (2014). Demonstrating kHz Frequency Actuation for Conducting Polymer Microactuators. Adv. Funct. Mater. 24 (30), 4851–4859. 10.1002/adfm.201400373

[B46] Merrill D. R. Bikson M. Jefferys J. G. R. (2005). Electrical Stimulation of Excitable Tissue: Design of Efficacious and Safe Protocols. J. Neurosci. Methods 141 (2), 171–198. 10.1016/j.jneumeth.2004.10.020 15661300

[B47] Nguyen-Vu T. D. Chen H. Cassell A. M. Andrews R. Meyyappan M. Li J. (2006). Vertically Aligned Carbon Nanofiber Arrays: an advance toward Electrical-Neural Interfaces. Small 2 (1), 89–94. 10.1002/smll.200500175 17193561

[B48] Nicolelis M. A. L. Dimitrov D. Carmena J. M. Crist R. Lehew G. Kralik J. D. (2003). Chronic, Multisite, Multielectrode Recordings in Macaque Monkeys. Proc. Natl. Acad. Sci. 100 (19), 11041–11046. 10.1073/pnas.1934665100 12960378 PMC196923

[B49] Nimbalkar S. Castagnola E. Balasubramani A. Scarpellini A. Samejima S. Khorasani A. (2018). Ultra-capacitive Carbon Neural Probe Allows Simultaneous Long-Term Electrical Stimulations and High-Resolution Neurotransmitter Detection. Sci. Rep. 8 (1), 1–14. 10.1038/s41598-018-25198-x 29725133 PMC5934383

[B50] Obien M. E. J. Deligkaris K. Bullmann T. Bakkum D. J. Frey U. (2015). Revealing Neuronal Function through Microelectrode Array Recordings. Front. Neurosci. 8, 423. 10.3389/fnins.2014.00423 25610364 PMC4285113

[B51] Ouyang L. Wei B. Kuo C. C. Pathak S. Farrell B. Martin D. C. (2017). Enhanced PEDOT Adhesion on Solid Substrates with Electrografted P(EDOT-NH2). Sci. Adv. 3 (3), e1600448. 10.1126/sciadv.1600448 28275726 PMC5336355

[B52] Polikov V. S. Tresco P. A. Reichert W. M. (2005). Response of Brain Tissue to Chronically Implanted Neural Electrodes. J. Neurosci. Methods 148 (1), 1–18. 10.1016/j.jneumeth.2005.08.015 16198003

[B53] Rasheed A. Howe J. Y. Dadmun M. D. Britt P. F. (2007). The Efficiency of the Oxidation of Carbon Nanofibers with Various Oxidizing Agents. Carbon 45 (5), 1072–1080. 10.1016/j.carbon.2006.12.010

[B54] Rizzo J. F. Wyatt J. Loewenstein J. Kelly S. Shire D. (2003). Methods and Perceptual Thresholds for Short-Term Electrical Stimulation of Human Retina with Microelectrode Arrays. Invest. Ophthalmol. Vis. Sci. 44 (12), 5355–5361. 10.1167/iovs.02-0819 14638738

[B55] Samba R. Herrmann T. Zeck G. (2015). PEDOT-CNT Coated Electrodes Stimulate Retinal Neurons at Low Voltage Amplitudes and Low Charge Densities. J. Neural Eng. 12 (1), 016014. 10.1088/1741-2560/12/1/016014 25588201

[B56] Saunier V. Flahaut E. Blatché M.-C. Bergaud C. Maziz A. (2020b). Carbon Nanofiber-PEDOT Composite Films as Novel Microelectrode for Neural Interfaces and Biosensing. Biosens. Bioelectron. 165, 112413. 10.1016/j.bios.2020.112413 32729532

[B57] Saunier V. Flahaut E. Blatché M.-C. Bergaud C. Maziz A. (2020a). Microelectrodes from PEDOT-Carbon Nanofiber Composite for High Performance Neural Recording, Stimulation and Neurochemical Sensing. MethodsX 7, 101106. 10.1016/j.mex.2020.101106 33145183 PMC7591727

[B58] Schultz A. E. Kuiken T. A. (2011). Neural Interfaces for Control of Upper Limb Prostheses: the State of the Art and Future Possibilities. PM&R 3 (1), 55–67. 10.1016/j.pmrj.2010.06.016 21257135

[B59] Shin S. Kim J. Jeong J. Gwon T. M. Choi G. J. Lee S. E. (2016). High Charge Storage Capacity Electrodeposited Iridium Oxide Film on Liquid crystal Polymer-Based Neural Electrodes. Sens. Mater. 28 (3), 243–260.

[B60] Sparreboom M. van Schoonhoven J. van Zanten B. G. A. Scholten R. J. P. M. Mylanus E. A. M. Grolman W. (2010). The Effectiveness of Bilateral Cochlear Implants for Severe-To-Profound Deafness in Children. Otology & Neurotology 31 (7), 1062–1071. 10.1097/mao.0b013e3181e3d62c 20601922

[B61] Spira M. E. Hai A. (2013). Multi-electrode Array Technologies for Neuroscience and Cardiology. Nat. Nanotech 8 (2), 83–94. 10.1038/nnano.2012.265 23380931

[B62] Sridharan A. Muthuswamy J. (2021). Soft, Conductive, Brain-like, Coatings at Tips of Microelectrodes Improve Electrical Stability under Chronic, *In Vivo* Conditions. Micromachines 12 (7), 761. 10.3390/mi12070761 34203234 PMC8306035

[B63] Theodore W. H. Fisher R. S. (2004). Brain Stimulation for Epilepsy. Lancet Neurol. 3 (2), 111–118. 10.1016/s1474-4422(03)00664-1 14747003

[B64] Venkatraman S. Hendricks J. Richardson-Burns S. Jan E. Martin D. Carmena J. M. (2009). “PEDOT Coated Microelectrode Arrays for Chronic Neural Recording and Stimulation,” in 2009 4th International IEEE/EMBS Conference on Neural Engineering (Antalya, Turkey: IEEE), 383–386. 10.1109/ner.2009.5109313

[B65] Viswam V. Obien M. E. J. Franke F. Frey U. Hierlemann A. (2019). Optimal Electrode Size for Multi-Scale Extracellular-Potential Recording from Neuronal Assemblies. Front. Neurosci. 13, 385. 10.3389/fnins.2019.00385 31105515 PMC6498989

[B66] Vitale F. Summerson S. R. Aazhang B. Kemere C. Pasquali M. (2015). Neural Stimulation and Recording with Bidirectional, Soft Carbon Nanotube Fiber Microelectrodes. ACS nano 9 (4), 4465–4474. 10.1021/acsnano.5b01060 25803728

[B67] Wang K. Chung-Chiun Liu C.-C. Durand D. M. (2009). Flexible Nerve Stimulation Electrode with Iridium Oxide Sputtered on Liquid crystal Polymer. IEEE Trans. Biomed. Eng. 56 (1), 6–14. 10.1109/tbme.2008.926691 19224713 PMC2738844

[B68] Wang K. Frewin C. L. Esrafilzadeh D. Yu C. Wang C. Pancrazio J. J. (2019). High‐Performance Graphene‐Fiber‐Based Neural Recording Microelectrodes. Adv. Mater. 31 (15), 1805867. 10.1002/adma.201805867 30803072

[B69] Wu X. Pei W. Zhang H. Chen Y. Guo X. Chen H. (2015). Sodium Dodecyl Sulfate Doping PEDOT to Enhance the Performance of Neural Microelectrode. J. Electroanalytical Chem. 758, 26–32. 10.1016/j.jelechem.2015.10.005

[B70] Zhou H. Cheng X. Rao L. Li T. Duan Y. Y. (2013). Poly(3,4-ethylenedioxythiophene)/multiwall Carbon Nanotube Composite Coatings for Improving the Stability of Microelectrodes in Neural Prostheses Applications. Acta Biomater. 9 (5), 6439–6449. 10.1016/j.actbio.2013.01.042 23402765

