# Supplementary material for: Nanofibrous PEDOT-Carbon Composite on Flexible Probes for Soft Neural Interfacing
Source: Front Bioeng Biotechnol. 2021 Nov 26;9:780197. doi: 10.3389/fbioe.2021.780197 (PMC8662776; doi:10.3389/fbioe.2021.780197)
Supplement: Supplementary file 1 [file DataSheet1.docx]

**Supplementary Information**

**Nanofibrous PEDOT-Carbon Composite on Flexible Probes for Soft Neural Interfacing**

*Venkata Suresh Vajrala^a^, Valentin Saunier^a^, Lionel G Nowak^,b^, Emmanuel Flahaut^c^, Christian Bergaud^a^, and Ali Maziz^a^**

* Corresponding authors: Dr. Ali MAZIZ, LAAS-CNRS, [ali.maziz@laas.fr](mailto:ali.maziz@laas.fr)

a Laboratory for Analysis and Architecture of Systems, CNRS, Toulouse, France

b Centre de Recherche Cerveau et Cognition (CerCo), CNRS, Toulouse, France

c CIRIMAT, Université de Toulouse, CNRS, route de Narbonne, F-31062 Toulouse, France


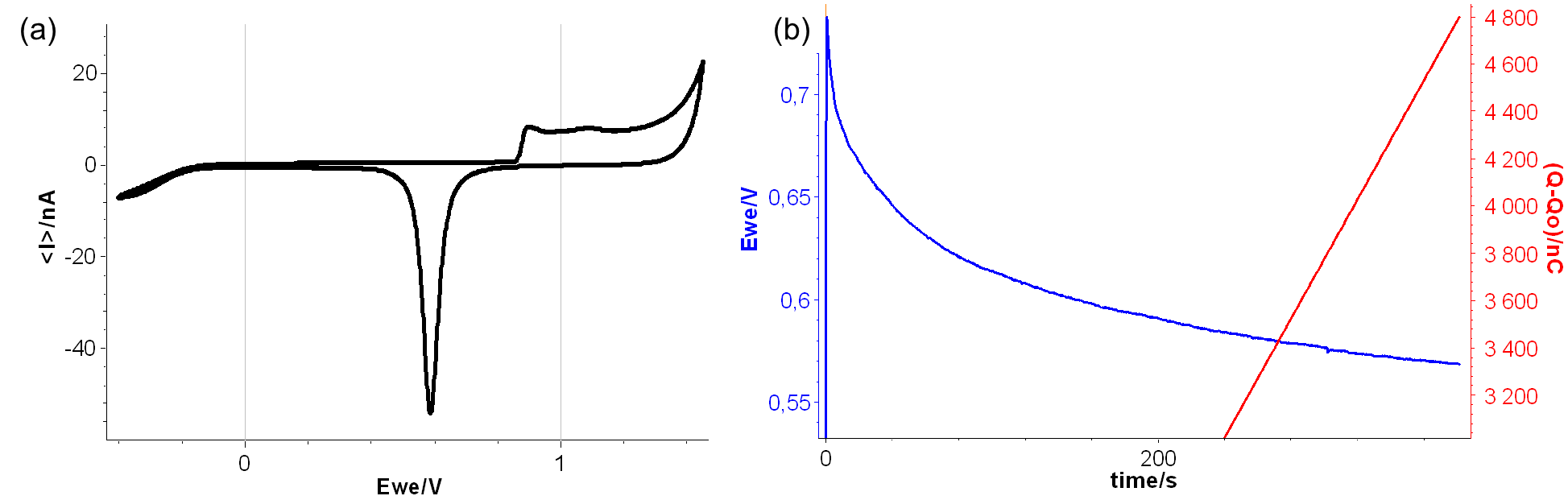
Figure S1: (a) Representative cyclic voltammetry of a clean gold electrode on flexible neural probe in 0.5M H_2_SO_4_ solution at a scan rate of 200 mV/sec *vs* Ag/AgCl reference. (b) Chrono-potentiometric curves obtained with flexible neural probe during the deposition of PEDOT-CNF composite on the gold surface at a deposition rate of 6 nC/µm^2^.


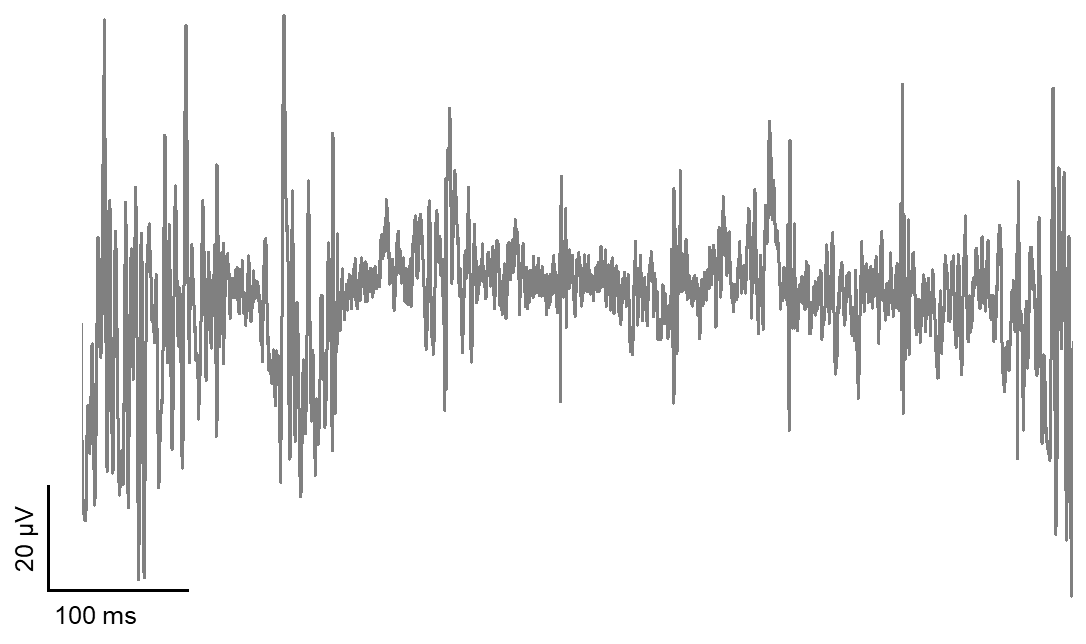


Figure S2: Representative signal obtained by a flexible gold microelectrode when placed in the hippocampal region (CA1 and CA3) of a mouse brain slice. A frequency filter (300 – 3000 Hz) was used here to obtain the resultant signal.

| Electrode  material | Flexible Substrate | Electrode surface area (µm²) | Imp K (Mohms.µm^2^) | CIL (mC/cm^2^) | Electrolyte | Ref |
| --- | --- | --- | --- | --- | --- | --- |
| PEDOT :Nafion/Au | Polyimide | 61575 | 97.5 | 4.5 | NaCl | [1] |
| PEDOT :PSS/ Au | Polyimide | 61575 | 121 | 3.5 | NaCl | [1] |
| PDMAAp/PEDOT/ IrOx-Pt | Polyimidie | 1000 | 10 | 3.7 | PBS | [2] |
| PEDOT :PSS-rGO | Au-wire/epoxy | 7850 | 59 | 6.9 | PBS | [3] |
| PEDOT-CNF | **Parylene** | **1250** | **16.8 ± (2)** | **7.6 ± (1.3)** | **aCSF** | */* |
| PtIr wires | xx | 17000 | 452 | 0.15 | PBS | [4] |
| Glassy Carbon/ Pt | Polyimide | 70685 | 410 | 3.5 | PBS | [5] |
| Porous graphene | Polyimide | 62500 | 125 - 500 | 3.1 | PBS | [6] |
| Graphene fiber wire | Parylene | 169 - 750 | 8.7 – 28.4 | 8.9 – 4.7 | PBS | [7] |
| CNT fiber wire | polyimide | 1450 | 20.5 | 6.5 | PBS | [4] |
| Sputtered IrOx/Ti | Liquid crystal polymer (LCP) | 1000000 | 0.4 | 4.6 | Phosphate/  bicarbonate buffer | [8] |
| Electrodeposited IrOx | LCP | 70685 | 100 | 1.3 | PBS | [9] |

Table S1: Comparison of the electrochemical performances of our PEDOT-CNF composite with few of the well-known materials to fabricate flexible neural interfacing microelectrodes.

**References:**

[1] S. Carli, M. Bianchi, E. Zucchini, M. Di Lauro, M. Prato, M. Murgia, et al., Electrodeposited PEDOT: Nafion composite for neural recording and stimulation, Advanced healthcare materials, 8(2019) 1900765.

[2] C. Kleber, K. Lienkamp, J. Rühe, M. Asplund, Wafer‐Scale Fabrication of Conducting Polymer Hydrogels for Microelectrodes and Flexible Bioelectronics, Advanced Biosystems, 3(2019) 1900072.

[3] S. Lee, T. Eom, M.-K. Kim, S.-G. Yang, B.S. Shim, Durable soft neural micro-electrode coating by an electrochemical synthesis of PEDOT: PSS/graphene oxide composites, Electrochimica Acta, 313(2019) 79-90.

[4] F. Vitale, S.R. Summerson, B. Aazhang, C. Kemere, M. Pasquali, Neural stimulation and recording with bidirectional, soft carbon nanotube fiber microelectrodes, ACS nano, 9(2015) 4465-74.

[5] S. Nimbalkar, E. Castagnola, A. Balasubramani, A. Scarpellini, S. Samejima, A. Khorasani, et al., Ultra-capacitive carbon neural probe allows simultaneous long-term electrical stimulations and high-resolution neurotransmitter detection, Scientific reports, 8(2018) 1-14.

[6] Y. Lu, H. Lyu, A.G. Richardson, T.H. Lucas, D. Kuzum, Flexible neural electrode array based-on porous graphene for cortical microstimulation and sensing, Scientific reports, 6(2016) 1-9.

[7] K. Wang, C.L. Frewin, D. Esrafilzadeh, C. Yu, C. Wang, J.J. Pancrazio, et al., High‐Performance Graphene‐Fiber‐Based Neural Recording Microelectrodes, Advanced Materials, 31(2019) 1805867.

[8] K. Wang, C.-C. Liu, D.M. Durand, Flexible nerve stimulation electrode with iridium oxide sputtered on liquid crystal polymer, IEEE transactions on biomedical engineering, 56(2009) 6-14.

[9] S. Shin, J. Kim, J. Jeong, T.M. Gwon, G.J. Choi, S.E. Lee, et al., High charge storage capacity electrodeposited iridium oxide film on liquid crystal polymer-based neural electrodes, Sens Mater, 28(2016) 243-60.
